# Longitudinal Pathogenesis Study of Young Red Deer (*Cervus elaphus*) after Experimental Challenge with *Mycobacterium avium* subsp. *paratuberculosis* (MAP)

**DOI:** 10.1155/2012/931948

**Published:** 2012-06-06

**Authors:** Colin Mackintosh, Gary Clark, Brendan Tolentino, Simon Liggett, Geoff de Lisle, Frank Griffin

**Affiliations:** ^1^AgResearch Invermay, P.O. Box 50034, Mosgiel, New Zealand; ^2^14 Rimu Lane, Wanaka 9305, New Zealand; ^3^Disease Research Laboratory, University of Otago, P.O. Box 56, Dunedin, New Zealand; ^4^AgResearch Wallaceville, P.O. Box 40063, Upper Hutt, New Zealand

## Abstract

Paratuberculosis progresses more quickly in young red deer than in sheep or cattle. This study describes the clinical, immunological and pathological changes over a 50-week period in fourteen 4-month-old red deer that received heavy oral challenge with *Mycobacterium avium* subsp. *paratuberculosis* (MAP). At 4 and 12 weeks post challenge they were anaesthetized and a section of jejunal lymph node was surgically removed for culture, histopathology, and genetic studies. All 14 deer became infected, none were clinically affected, and they had varying degrees of subclinical disease when killed at week 50. Week 4 biopsies showed no paratuberculosis lesions, but MAP was cultured from all animals. At weeks 12 and 50 histopathological lesions ranged from mild to severe with corresponding low-to-high antibody titres, which peaked at 12–24 weeks. IFN-**γ** responses peaked at 8–15 weeks and were higher in mildly affected animals than in those with severe lesions.

## 1. Introduction

Paratuberculosis (Johne's disease), caused by *Mycobacterium avium* subsp. *paratuberculosis* (MAP), can cause significant loss of production in red deer (*Cervus elaphus*) on farms in New Zealand [1, 2]. Young deer are particularly susceptible [3] and clinical disease often occurs in 8–27-month-old animals [1], compared with 2–4 years in sheep and cattle. Clinically affected young deer typically have diarrhoea, lose weight, and have some thickening of the small intestines and grossly enlarged mesenteric lymph nodes, often with tuberculosis-like caseous lesions [2].

Although early immune responses have been studied in cattle [4, 5] and in sheep [6], there is little published information about the progression of disease and immunological responses over the 12 months after challenge with MAP in red deer. Early innate and acquired immune responses are thought to play important roles in determining the pathogenesis and severity of the disease.

An experimental paratuberculosis challenge model for red deer has been developed to reproduce a typical range of disease outcomes from mild subclinical infection to clinical disease [7]. This challenge model has been used to study the dose response and differences in susceptibility of deer to ovine and bovine strains of MAP [8], protective efficacy of vaccines [9], and age susceptibility [3].

The two main objectives of this study were to monitor the clinical, immunological, and pathological changes over the course of a year and to collect biopsy samples of jejunal lymph node at three time points for future study of gene expression related to resistance/susceptibility MAP.

## 2. Materials and Methods

### 2.1. Animals

Seventeen red deer calves were born to 22 randomly chosen mixed-age seronegative hinds that had been inseminated with frozen semen from two unrelated red stags (7 to sire 1, 10 to sire 2) on a farm which had no previous history of clinical paratuberculosis and has been tested free of bovine tuberculosis for 30 years. These calves were weaned in late February when they were ~3 months of age. They were treated in early March with pour-on moxidectin (Cydectin; Fort Dodge Animal Health Ltd., Auckland, NZ), dosed with copper oxide wire particles (4 g Copper Capsule; Bayer NZ Ltd., Auckland, NZ), and vaccinated with Yersiniavax (AgVax Developments Ltd., Upper Hutt, NZ). This study was approved by the AgResearch Invermay Ethics Committee (AEC 11425).

### 2.2. Challenge

 In late March 2008, the 17 deer, which were all Paralisa test negative, were challenged with MAP using a standard infection model described previously [7, 8]. Briefly, each animal received an oral challenge dose daily for four days of ~10^9^ cfu of a virulent bovine strain of MAP harvested directly from the jejunal lymph nodes (JJLNs) of clinically affected deer. The estimated number was based on BACTEC 12B liquid culture of serial dilutions.

### 2.3. Clinical Monitoring

The deer were grazed together at pasture throughout the 50-week study and their diet was supplemented with hay and barley over the winter. Their physical condition was monitored daily and they were weighed monthly in April, May, and June, two weekly in July and weekly from August to early March. The protocol required that any deer that developed early clinical signs of paratuberculosis (loss of 5–10% body weight over a two week period, loss of muscle mass, ± diarrhoea) would be euthanised.

### 2.4. Jejunal Lymph Node Sampling

At 4 and 12 weeks post challenge (pc) each deer was anaesthetized using intravenous fentanyl citrate, xylazine, and azaperone (Fentazin 5; Bomac Laboratories Ltd., Manukau), intubated and maintained on halothane (Halothane-Vet; Merial Ancare, Manukau) and oxygen. The animal was placed in dorsal recumbency and the abdomen was clipped, prepped, and draped. A 15 cm long midline laparotomy was performed and the gross appearance of the intestines, mesenteric lymph nodes, and mesenteries noted. A 40–50 mm piece of posterior JJLN was excised and cut into five pieces; three pieces were placed in cryotubes and immediately frozen in liquid nitrogen for future gene expression studies, one piece was fixed in 10% buffered formalin for histopathological examination, and one piece was cultured for MAP. The incision was closed with Maxon sutures (Davis & Geck, USA) in the linea alba and Vicryl sutures (Ethicon, USA) in the skin. The animals were injected with long-acting penicillin (Norocillin LA; Norbrook NZ Ltd., Rangiora) to prevent postoperative infection and with meloxicam (Metacam 20; Boeringer Ingelheim NZ Ltd., Auckland) for postoperative pain relief. The anaesthetic was reversed with yohimbine and naloxone (Contran H; Bomac Laboratories Ltd., Manukau). The deer were monitored closely for two weeks following each biopsy session.

All surviving deer were euthanised 50 weeks pc and necropsied and samples were taken from the anterior, mid, and posterior jejunum (JJ), ileocaecal valve (ICV), and associated lymph nodes and processed as for the biopsies. At biopsy and necropsy any gross lesions of paratuberculosis in the JJ, ICV, JJLN, ileocaecal lymph node (ICLN), and mesenteries were described and graded according to the following gross lesion scores: 0, no visible lesions (NVLs); 1, slightly enlarged JJLN/ICLN; 2, moderately enlarged JJLN/ICLN; 3, very enlarged JJLN/ICLN; 4, enlargement plus a single caseogranulomatous JJLN or ICLN lesion; and 5, multiple JJLN and ICLN lesions.

### 2.5. Immune Monitoring

Blood samples were taken at regular intervals during the trial and tested with the Paralisa test and gamma interferon (IFN-*γ*) assay and a comparative cervical tuberculin test (CCT) was carried out two weeks prior to slaughter. The Paralisa test is an IgG_1_ antibody class-specific ELISA test using two antigens, namely, MAP protoplasmic antigen (PPA) and MAP purified protein derivative (PPDJ) and uses an in-house anti-red deer antibody [10, 11]. The results are expressed as ELISA units (EUs) calculated by (OD_sample_ − OD_neg control_) × 100 and if an animal has a titre >50 EU to either antigen, it is considered positive. The IFN-*γ* assay uses an in-house ELISA assay [12] to measure IFN-*γ* responses in heparinised blood samples stimulated with PPDj. Briefly, 96-well flat-bottomed microtiter plates were coated with IFN-*γ* capture antibody (Serotec). Wash buffer only was added as a negative control, and recombinant cervine IFN-*γ* was added as a positive control. The detection system used biotinylated antibody (Serotec) and streptavidin-horseradish peroxidase conjugate. Plates were read in an ELISA reader at 450 nm with reference at 650 nm. The results were expressed as (PPDj − neg control) in ELISA units. For the CCT, intradermal injections of 0.1 mL of avian tuberculin (2500 IU) and bovine tuberculin (5000 IU) were given at two closely clipped sites on the neck. Skin thickness measurements were taken before injection and ~72 hours later and the increase in skin thickness was calculated.

### 2.6. Culture

 All tissue and faecal samples (5 g) were cultured for MAP using BACTEC 12B liquid culture medium containing egg yolk and mycobactin [13, 14], which gives semiquantitation according to the speed at which the samples turn positive; that is, the number of days to positive (dtp), with the cultures read every 5–7 days.

The JJLN biopsy samples taken at week 4 and week 12 pc were chilled and cultured within 48 hours of collection. At week 50 a pool of four intestinal samples (anterior, mid and posterior JJ and ICV) and a pool of four corresponding lymph node samples (JJLN and ICLN) from each animal were collected immediately after death, chilled overnight, and cultured the next day. Faecal samples collected from the rectum at week 50 pc were chilled overnight, processed using the double incubation method [15], and then cultured.

### 2.7. Histopathology

Sections of fixed samples from each animal were stained with H&E and Ziehl-Neelsen (ZN) and the histopathological lesion severity scores (LSSs) were assessed using a modified 7-point scale for both the mesenteric lymph nodes (MLN) and the enteric lesions. The week 50 MLN and enteric scores were added together for a total LSS score on a 14-point scale [16, 17]. Sections were examined without knowledge of animal breeding or gross lesions. For MLN and enteric lesions, LSSs of 1 and 2 were regarded as very mild or nonspecific, 3 as suggestive of very mild paratuberculosis, 4 as mild, 5 as moderate, 6 as moderately severe, and 7 as severe paratuberculosis. For the total LSS additive scores of 4–8 were mild, 9–11 were moderate, and 12–14 were severe paratuberculosis. Lesions were also described as no acid-fast organisms (AFOs), paucibacillary (PB), or multibacillary (MB) [16].

### 2.8. Statistical Analysis

 There were insufficient animals in this study for meaningful statistical analysis.

## 3. Results

### 3.1. Clinical Outcomes

No animals showed signs of clinical paratuberculosis and the deer all gained weight throughout the study, with hinds averaging 35 kg and stags 56 kg weight gain over the 50 week study, and there was no significant difference in weight gain between Sire 1 and Sire 2 offspring. Three deer died of misadventure unrelated to paratuberculosis.

### 3.2. Gross Lesion Score

There were no gross lesions apparent in the JJLN at week 4, but by week 12 half the deer had slightly or moderately enlarged JJLN (Table 1). At week 50 four deer had very enlarged JJLN and one of these animals had multiple caseous foci, while the other ten deer had no or slight enlargement of the JJLN.

### 3.3. Histopathological Lesion Severity Score

There were no specific lesions seen at Week 4, but by Week 12 MLN LSSs ranged from mild to severe (4–7) (Table 1), although these were scored conservatively because they were based only on a small piece of posterior JJLN. At Week 50 MLN LSSs ranged from 2 to 7, and total LSS, from 2 to 14. One animal had no visible AFO, three had no AFO but were classed as PB, one was MB, and the rest were PB.

Over the period from Week 12 to Week 50 the following changes in MLN LSS occurred; 11 of 14 reduced, one increased and two remained the same. Of those that became less severe, one went from severe to very mild, two from severe to mild, three from severe to moderate, one from severe to moderately severe, one from moderate to mild/nonspecific, two from moderate to mild, and one mild went to nonspecific. The one that increased in severity went from mild to moderate. One severe and one moderately severe remained the same. At Week 50, MLN and enteric LSS generally had similar scores.

Interestingly, an increased concentration of AFO in calcified tissue present in two of the animals at Week 50 was associated with PB, as per criteria described by Clark et al. in 2010 [16]. The same animals were MB at week 12.

There was no apparent difference in LSS, the changes in LSS with time or PB/MB ratio between offspring of the two sires throughout the study.

### 3.4. Culture

MAP was isolated from all JJLN biopsies at weeks 4 and 12 and from pooled LNs at Week 50 of all 14 deer (Table 2). Culture times for JJLN of 28–42 dtp at Week 4 suggest small numbers of MAP present, but by Week 12 this had reduced to 12–21 dtp, indicating an increase in numbers of MAP, with the three least affected animals averaging 19 dtp compared with 13 dtp for the most severely affected. By Week 50, the least affected had increased to 31 dtp, and the worst affected increased slightly to 18 dtp. At Week 50 the dtp for JJ + ICV tended to be higher than that for JJLN + ICLN. At slaughter only 4/14 were faecal-culture positive (31–37 dtp) indicating relatively low numbers of MAP present.

### 3.5. Serology

**Figure 1:**
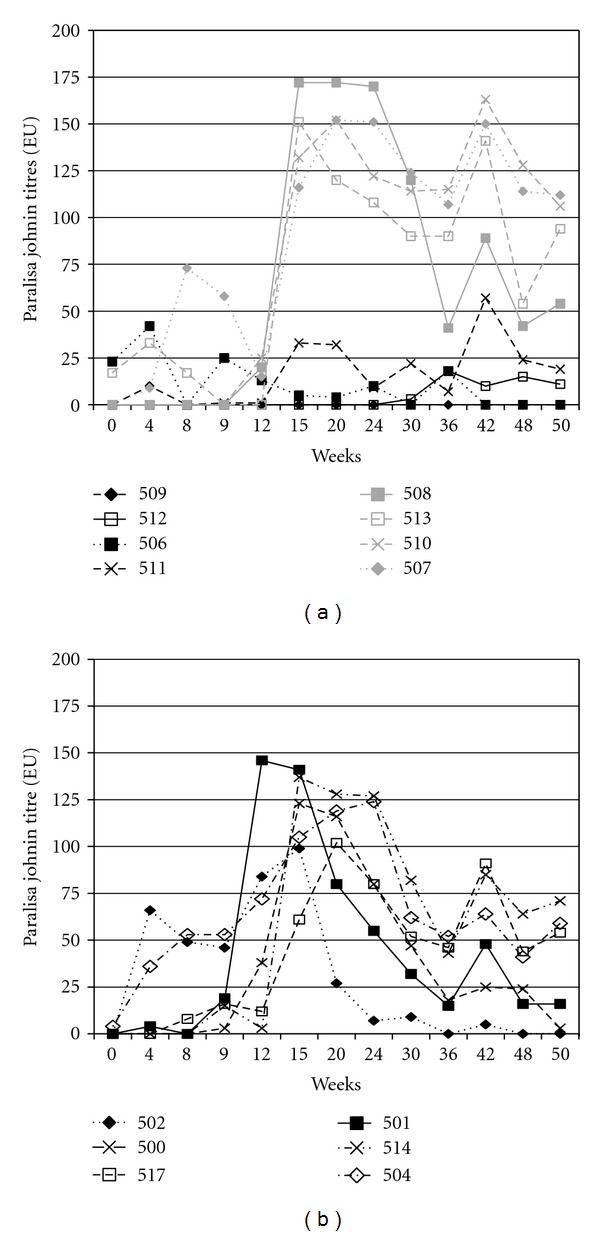
(a) Paralisa titres (PPA antigen) in ELISA units (EU) over the 50 week study for the four deer (506, 509, 511, 512) that had the lowest serological response to the Paralisa Johnin antigen (black lines), and the four deer (507, 508, 510, 513) that had the highest and most sustained serological response to Johnin (gray lines). (b) Paralisa titres (PPA antigen) in ELISA units (EU) over the 50 week study for the six deer that had an intermediate serological response to the Paralisa Johnin antigen.

Over the course of the 50 week study, four of the animals (506, 509, 511, and 512) had low titres to Johnin throughout and all peaked at <60 EU (Figure 1(a)) and three of these had mild-to-moderate lesions (total LSS 2–8). By contrast, four animals (508, 513, 510, and 507) had high titres 151–172 EU that peaked 15–20 weeks pc and tended to remain high (Figure 1(a)) and had moderate-to-severe lesions (total LSS 8–12). The remaining six animals had intermediate titres 99–146 EU that peaked 12–26 weeks pc and tended to decline to much lower levels (Figure 1(b)) and had mild-to-severe lesions (total LSS 6–12). Animals with higher titres (504, 508, 510, 514, and 517) tended to rise at Week 50, two weeks after the skin test at Week 48, while animals with the lowest titres (500, 501, 502, 506, 509, 511, 512) do not rise.

### 3.6. IFN*γ*

**Figure 2:**
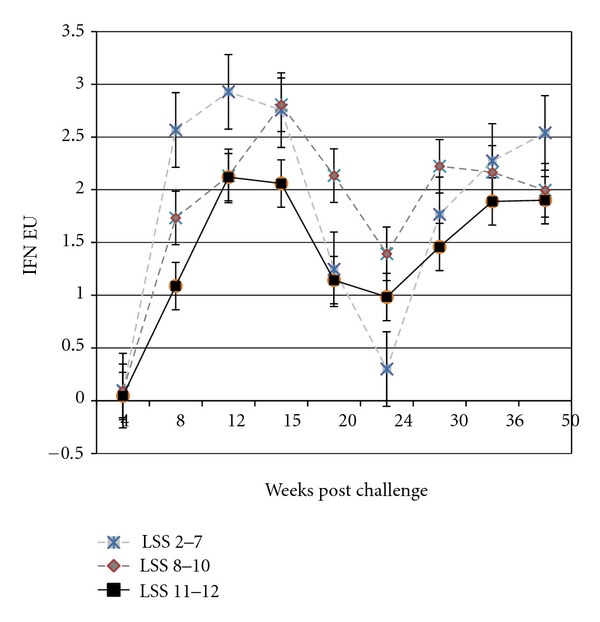
Mean gamma interferon (IFN-*γ*) assay results in ELISA units (EUs) for the 14 deer sorted into three groups based on their total lesion severity score (LSS) at week 50 after MAP challenge at day 0.

The IFN*γ* of the least affected animals (total LSS 2–7) rose earlier and peaked higher than those of the more severely affected animals (total LSS 11-12) during the 50-week study (Figure 2). The IFN*γ* responses peaked twice, with the first peak at 12–15 weeks pc, a decline to a low at 24 weeks, and a second peak at 30–36 weeks. Only the LSS 2–7 group had an increase in IFN*γ* at Week 50, 2 weeks after the skin test.

### 3.7. CCT

 The increase in skin thickness at the avian and bovine sites averaged 9.7 mm (1.8–13.4) and 3.1 mm (0.6–8.4), respectively, with *A* > *B* in all animals; that is, negative for bovine Tb. The average avian site increase for the three most severely affected deer was 8.2 mm (6.5–10) compared with 3.5 mm (1.8–5) for the least affected.

## 4. Discussion

The red deer MAP infection model allowed a number of variables to be controlled so that their immune response to challenge could be studied efficiently. The offspring were all the same age, having been bred on the same day by artificial insemination of synchronised hinds, which calved within a few days of each other. The animals were all run together in one mob from birth, treated regularly with anthelmintics, vaccinated against yersiniosis, treated with mineral supplements, grazed at pasture, and given the same infective dose of MAP on the same days. The only common variable that was not controlled was gender, but there was no obvious gender bias in the results. In previous studies a proportion of young red deer challenged by MAP have developed clinical disease [3, 8, 9]. In this study, although the age of animal and the strain of MAP were the same as previously, there were no clinically affected animals and few had severe disease, suggesting that the offspring of both sires had intermediate resistance to MAP, or the inoculum had fewer MAP than previously.

The use of the infection model together with the sampling of jejunal lymph nodes at 4, 12, and 50 weeks pc and periodic serology and IFN-*γ* assays enabled us to gain insights into the pathogenesis of MAP infection as well as longitudinal information about the immune responses. Deer appear to be relatively susceptible to paratuberculosis and the disease progresses much more quickly in susceptible deer than that in sheep and cattle. Under natural farm conditions, clinical cases commonly occur in red deer between 8 and 15 months of age [1], whereas clinical disease is more commonly seen in sheep and cattle aged 3-4 years, which make natural disease harder to study in a reasonable time frame. The scoring of lesion severity [16, 17] based on histopathological examination of biopsy and necropsy samples, together with MAP culture times, enabled objective longitudinal observations of disease within and between animals over time. The scoring of lesion severity for the biopsy samples at Weeks 4 and 12 was conservative because it was only based on a single piece of posterior JJLN, whereas the samples taken at necropsy were based on four samples of anterior, mid, and posterior JJ and ICV plus their associated lymph nodes.

There have been a small number of studies in sheep and cattle that have used surgical biopsies of small intestine and mesenteric lymph nodes to diagnose paratuberculosis [18–20] or to follow the course of infection and to attempt to associate these changes with immunological measurements [6, 21, 22]. Gilmour et al. biopsied five experimentally infected Cheviot x Suffolk sheep on four occasions 5, 11, 17, and 23 months and at necropsy 27 months pc while Dennis et al. monitored a total of 77 naturally infected Merino sheep and biopsied 20 of these at 12, 18, and 24 months of age. Clinically affected sheep were euthanised and necropsied, as were all the remaining sheep 36 months of age. Gilmour et al. also killed groups of sheep at intervals and necropsied them as well as clinically affected animals. Both these groups obtained a wide range of disease outcomes over a 27–36-month period. Wu et al. infected one-month-old calves and then monitored disease progression by taking biopsies of the small intestine and mesenteric lymph node at 1-2-month interval and calves were killed at 6–9 months of age. There were no signs of clinical disease, although they detected changes in the inflammatory responses and the levels of cytokines indicated Th1-type-associated cellular, but not Th2-type-associated humoral responses. In contrast the deer model used in this study has the advantage of reproducing a range of natural disease outcomes in a one-year study.

An interesting outcome of this study was the spectrum of changes in disease severity that took place over the course of a year. At Week 4 MAP was cultured from the JJLN of all animals but there were no paratuberculosis-specific lesions seen on histology. Between Weeks 4 and 12 all the deer showed an increase in MAP numbers and they developed JJLN lesions ranging from mild to severe. However, MAP numbers increased more in the animals that went on to become more severely affected, suggesting that differences in relative resistance/susceptibility were influencing the development of lesions and rate of MAP multiplication. Between Week 12 and Week 50, some animals remained mildly affected, some remained severely affected, while some improved and others got worse. About half the animals managed to suppress the multiplication of MAP and limit the extent of disease, such that the LSS and the number of MAP in the JJLN declined, as indicated by an increase in dtp. In contrast, some of the animals appeared to be unable to suppress MAP multiplication, their LSS worsened, and number of MAP present increased or stayed the same over this 9-month period. Two animals that were MB at Week 12 were found to be PB at Week 50 but had a few foci of high numbers of AFO in mineralized tissue. This type of lesion has been observed previously in naturally infected animals [16] and the pathogenesis appears to relate to a previous MB lesion phase, which is a new finding.

The most severely affected animals also had the highest antibody titres, while the two least affected animals remained seronegative throughout the study and had higher earlier IFN-*γ* levels than the more severely affected animals (LSS 5–7). These findings support the view that the more resistant animals have a more effective Th1 cell-mediated immune response, while the more susceptible animals switch to a Th2 humoral response earlier [23]. The results suggest that innate immunity may have played only a minor role in the killing or suppression of multiplication of MAP in the first 4 weeks pc and that acquired immunity played a major role in the more resistant animals. It is interesting to note that even the relatively resistant animals still took some months to get on top of the infection and to reduce the number of MAP as well as lessen the apparent severity of the lesions. It is likely that the migration of infected macrophages and dendritic cells from the small intestine to the draining lymph node is essential for efficient local T-cell activation in paratuberculosis, in the same way that dendritic cell migration from the lungs to the draining lymph nodes is thought to be critical in T-cell activation in TB [24]. This appears to have occurred within a few weeks of MAP challenge in deer. It was not practical in this study to biopsy the small intestine at the same time as the JJLN, but monitoring the changes in the JJLN gave useful longitudinal insight into the immunological and pathological changes over the 50 weeks.

Similar observations have recently been reported in naturally infected sheep that were monitored over a 36-month period using lymph node and intestine biopsies [6]. Sheep that developed severe multibacillary enteritis never improved over time, and the affected sheep expressed clinical signs within 12 months of showing these severe lesions. However, six of the 46 (8.7%) sheep that had MAP culture positive biopsies at some stage, later had negative cultures at necropsy, suggesting recovery from infection. The study also showed that infection was never detected in 40.3% of the sheep in this flock, which were exposed to heavy natural challenge, suggesting that some animals were resistant to infection.

The longitudinal changes in lesion severity and number of MAP in this study are similar although not as extreme as those seen in a subsequent study undertaken in 2009, which also showed significant heritable resistance/susceptibility to experimental paratuberculosis challenge in the offspring of two sires that had displayed differences in resistance/susceptibility to paratuberculosis [25].

## 5. Conclusions

The longitudinal monitoring of histopathology and culture of the jejunal lymph nodes, as well as serology and IFN-*γ* responses, have given some insight into the pathogenesis of subclinical paratuberculosis in young red deer. Heavy oral challenge resulted in all animals becoming infected, but the course of infection varied greatly. MAP numbers increased in all animals up to 12 weeks pc and MLN lesions were mild to severe. However, MAP numbers increased more in the animals that went on to become more severely affected, suggesting that differences in relative resistance were influencing the development of lesions and rate of MAP multiplication. Between Week 12 and Week 50, half the animals managed to largely suppress the multiplication of MAP and limit the amount of disease, while others appeared unable to suppress MAP multiplication and their lesions became more severe. The most severely affected animals also had the highest antibody titres, while the two least affected animals remained seronegative throughout the study. This, together with the observation that the least affected animals had earlier and higher IFN-*γ* responses, supports the view that the more resistant animals had a more effective Th1 cell-mediated immune response. Innate immunity did not prevent infection in any of these animals. However, the decline in the severity of disease between 12 and 50 weeks pc suggests that acquired immunity played a major role in the more resistant animals.

## Figures and Tables

**Table 1:** Gross lesion score (Weeks 12 and 50), histopathological lesion severity scores (LSSs) for mesenteric lymph node (MLN), enteric (ENT) and total, and description of acid fast organisms (AFO) in the lesions as none, paucibacillary (PB), or multibacillary (MB) for samples biopsied at weeks 4 and 12 and taken at week 50 necropsy of the offspring of two unrelated sires.

Sire	Tag	Week 4		Week 12			Week 50				
		Sex	MLN LSS	Gross	MLN LSS	AFO	Gross	MLN LSS	ENT LSS	Total LSS	AFO
1	506	F	2	0	5	PB	0	2	0	2	None
1	511	F	0	0	4	PB	0	2	2	4	PB
1	513	M	0	2	7	MB	0	4	4	8	PB
1	508	F	0	2	7	MB	4	6	6	12	PB
1	507	M	0	2	7	MB	5	7	5	12	MB

2	514	M	0	2	7	PB	4	4	2	6	PB
2	504	F	0	1	7	PB	0	3	4	7	PB
2	509	M	0	0	5	PB	0	4	4	8	PB
2	502	M	0	2	7	PB	3	5	4	9	PB
2	501	M	0	0	7	MB	0	5	5	10	PB
2	500	F	0	0	5	MB	0	4	6	10	PB
2	512	M	0	0	5	MB	1	5	6	11	PB
2	510	M	2	2	7	MB	1	5	6	11	PB
2	517	F	0	0	5	MB	0	6	6	12	PB

Gross lesion scores: 0, no visible lesions (NVLs); 1, slightly enlarged jejunal lymph nodes (JJLNs) and/or ileocaecal lymph nodes (ICLNs); 2, moderately enlarged JJLN/ICLN; 3, very enlarged JJLN/ICLN; 4, enlargement plus a single caseogranulomatous JJLN or ICLN lesion; 5, multiple JJLN and ICLN lesions.

MLN and ENT LSSs of 1 and 2 were regarded as very mild nonspecific, 3 as suggestive of very mild paratuberculosis, 4 as mild, 5 as moderate, 6 as moderately severe and 7 as severe paratuberculosis. Lesions were also described as no AFO, paucibacillary (PB), or multibacillary (MB).

**Table 2:** Summary of BACTEC culture results in terms of the number of days to positive (dtp) for jejunal lymph node (JJLN) samples taken at Weeks 4 and 12, and for a pool of three samples of JJLN plus ileocaecal lymph node (ICLN) and a pool of three jejunum and ileo-caecal valve samples (JJ + ICV) and faecal samples (FSs) at week 50, compared with MLN lesion severity scores (LSSs) at each time point, with the animals sorted by total LSS at week 50 and designated as least or most affected.

Tag	Sire	Week 4		Week 12			Week 50			
		MLN LSS	JJLN	MLN LSS	JJLN	MLN LSS	JJLN + ICLN	JJ + ICV	FS	Affected
506	1	2	36	5	21	2	34	34	Neg	Least
511	1	0	36	4	21	2	34	34	Neg	Least
509	2	0	36	5	15	4	26	26	Neg	Least

Mean			**36**	5	**19**	**3**	**31**	**31**		

514	2	0	28	7	15	4	34	26	Neg	
504	2	0	32	7	15	3	26	21	Neg	
513	1	0	32	7	15	4	18	18	31	
502	2	0	36	7	12	5	18	26	Neg	
501	2	0	42	7	12	5	18	34	37	
500	2	0	35	5	12	4	21	18	Neg	
510	2	2	36	7	12	5	18	26	Neg	
512	2	0	36	5	12	5	18	34	31	

Mean			**35**	**6**	**13**	**5**	**21**	**25**	**33**	

517	2	0	36	5	12	6	18	26	Neg	Most
508	1	0	32	7	15	6	18	26	Neg	Most
507	1	0	36	7	12	7	18	18	37	Most

Mean			**35**	7	**13**	**6**	**18**	**23**	**37**	
